# Mississippi Falls in, Also

**Published:** 1903-05

**Authors:** 


Mississippi Falls In, Also. I reproduce the following from my sprightly young contemporary at Vicksburg—one of whose staff I knew when he was a baby. It is sensible and expresses my views on the subject. Let us *try*. Let us hope. Let there be "no such word as fail."

"The State Association has taken the radical step so long and earnestly considered in the adoption of the new constitution. It is now an affiliated organization and works under the laws and supervision of the American Medical Association. In the main, so far as the actual meetings of the association are concerned, the changes will not be greatly manifest except that the purely business transactions will no longer be brought before the main body but will be considered by the house of delegates, thereby facilitating dispatch and giving more time for scientific work. But the main features lie beneath the surface and go, or are intended to go, widely throughout the profession. The new movement is essentially one of organization and its object is to draw together medical men of the State and to weld them into one compact body. As the American Medical Association is to the State Association so the State Association is to its county societies. It encourages them, fosters them, sustains them, and in return will draw from them that pabulum of financial, mental and moral nature without which it itself can not exist. The work, therefore, is not of the "once a year" order but, to get the full benefit of the methods, must be carried into our county societies—must be lived out in our daily lives.

To the casual eye it seems at first glance decidedly visionary. The work of organizing the entire body of medical men, when our present State Association does not contain a quarter of their number, seems a herculean task indeed. The benefits to us as individuals from the workings of this vast machine are admittedly great, but so great as to appear in the nature of some fair "pipe dream." But it has been done elsewhere, why not with us? We refer the doubting Thomases who can see no possibility of success to the last Transactions of the Alabama State Medical Association and beg to remind them that "what man has done man may do."—*Mississippi Medical Record.*

